# Semiparametric discovery and estimation of interaction in mixed exposures using stochastic interventions

**DOI:** 10.1515/jci-2024-0058

**Published:** 2026-01-19

**Authors:** David B. McCoy, Alan Hubbard, Mark van der Laan, Alejandro Schuler

**Affiliations:** Division of Biostatistics, University of California, Berkeley, USA; Division of Biostatistics, University of California, Berkeley, USA.; Division of Biostatistics, University of California, Berkeley, USA.; Division of Biostatistics, University of California, Berkeley, USA.

**Keywords:** targeted maximum likelihood estimation, mixtures, interactions, ensemble learning, 62G05, 62G08, 62G20, 62P10

## Abstract

Understanding the complex interactions among multiple environmental exposures is critical for assessing their combined impact on health outcomes. This study introduces InterXshift, a novel semiparametric method that provides a nonparametric definition of interaction and facilitates both the discovery and efficient estimation of interaction effects in mixed exposures. Leveraging stochastic shift interventions and ensemble machine learning, InterXshift identifies and quantifies interactions through a model-independent target parameter, estimated using targeted maximum likelihood estimation (TMLE) and cross-validation. The approach contrasts expected outcomes from joint interventions against those from individual exposures, enabling the detection of synergistic and antagonistic interactions. Validation through simulations and application to the National Institute of Environmental Health Sciences (NIEHS) Mixtures Workshop data demonstrate InterXshift’s efficacy in accurately identifying true interaction directions and consistently highlighting significant impacts. We apply our methodology to National Health and Nutrition Examination Survey (NHANES) data to understand the interaction effect (if any) of furan exposure on leukocyte telomere length. This method enhances the analysis of multi-exposure interactions within high-dimensional datasets, offering robust methodological improvements for elucidating complex exposure dynamics in environmental health research. Additionally, we provide an opensource implementation of InterXshift in the InterXshift R package, facilitating its adoption and application by the research community.

## 1 Introduction

Understanding the combined effects of multiple exposures on health outcomes remains a pivotal challenge in environmental epidemiology. Researchers frequently seek to determine whether such exposures act *additively* or if they exhibit synergistic or antagonistic *interaction*. Classical approaches to interaction often focus on two binary treatments [1, 2], employing metrics like the relative excess risk due to interaction (RERI) to measure deviations from additivity. However, extending these concepts to multiple, *continuous* exposures – such as environmental pollutants – introduces substantial complexity, particularly when exposures may be correlated, nonlinearly associated with outcomes, or confounded by additional covariates.

### Connection to Prior Work on Additive Interaction.

While the concept of additive interaction has long been formalized in terms of risk difference and metrics such as the RERI [1, 2], these existing frameworks typically assume binary exposures and rely on parametric modeling. Our approach, in contrast, generalizes the RERI notion of “departure from additivity” to accommodate *continuous* exposures through shift interventions. This perspective preserves the fundamental interpretation of synergy or antagonism but embeds it in a semiparametric framework, allowing for partial or scaled exposure reductions. Further, whereas Vansteelandt et al. [1] introduced methods for multiply robust estimation of interaction parameters, we rely on nonparametric machine-learning algorithms for both outcome regression and exposure mechanisms to provide robustness and flexibility in high-dimensional settings. By recasting classical additive interaction ideas into a stochastic intervention framework, InterXshift unifies these influential concepts with modern data-adaptive causal inference techniques for continuous exposures.

### Methods Used in Environmental Mixtures.

The growing literature on environmental *mixtures* has highlighted both the importance and the difficulty of detecting and quantifying interaction effects. Traditional regression-based strategies that include pairwise product terms in generalized linear models may suffer from exponential growth in model parameters and from interpretability issues when numerous interactions are present. Recently developed approaches address mixture effects more flexibly: Bayesian kernel machine regression [3] can model nonlinearities without explicitly enumerating interactions, weighted quantile sum regression [4, 5] and quantile-based g-computation [5] handle correlated exposures in a more aggregated manner, and factor or clustering methods group exposures [6, 7]. Although these techniques can reveal overarching mixture signals, they are less suited for *systematically isolating* which exposure pairs are synergistic or antagonistic.

Several recent methods have advanced the state of the art in interaction detection. Forward selection algorithms [8] and machine-learning-based approaches [9] attempt to identify important nonlinear interactions among exposures. Bayesian factor analysis and Gaussian processes [10, 11] have been adapted to infer high-dimensional interactions, while others use semiparametric priors for mixtures [12, 13]. Notably, Chattopadhyay et al. [14] recently proposed Bayesian procedures to characterize synergy and antagonism in mixtures from a toxicological perspective, echoing classical notions of super-additive and sub-additive joint effects [15, 16]. However, many of these approaches either rely on specific model formulations or focus on measures (like posterior inclusion probabilities) that are not grounded in a direct, nonparametric *causal* definition of interaction.

### Limitations of Common Dimension-Reduction and Penalized Methods.

Other popular strategies reduce the dimensionality of an exposure set or impose sparsity in a regression framework but may overlook or conflate interactions on an *additive* outcome scale. For instance, principal component analysis (PCA) and related factor-based methods project exposures onto a small number of components, capturing broad variance structure but blending possible synergy signals into aggregate factors. Penalized regressions such as the LASSO can, in theory, detect pairwise interactions by including product terms, yet tuning choices and interpretability become challenging when many interaction terms are possible. Although such methods can aid in screening or dimension reduction, they do not yield a *target parameter* for synergy that directly represents additive departures from “no interaction.” Likewise, while Bayesian kernel machine regression (BKMR) flexibly models nonlinearities among multiple exposures, it does not inherently provide a causal, additive interaction parameter. In contrast, our proposed InterXshift approach defines and estimates a *stochastic*-*intervention*-based measure of synergy or antagonism, anchored in the *potential outcome* framework. This perspective facilitates clearer scientific interpretation – quantifying how a *joint shift* in two exposures modifies the outcome beyond their individual effects – and avoids the interpretability pitfalls or heavy modeling constraints often encountered in dimension-reduction or penalized regression approaches.

### Beyond Binary Exposures.

Within causal inference, it is well understood that interactions can be defined as *departures from additivity of causal effects* [1, 2]. For binary treatments, one can interpret additive interaction in terms of potential outcomes. Yet, for observational data with multiple continuous exposures, key questions arise: (i) how to specify a realistic intervention, and (ii) how to define synergy or antagonism on the outcome scale. A promising route is to replace the notion of “set A=a” with a *stochastic shift* in the distribution of A [17], enabling analyses of partial exposure reductions that resonate with practical regulatory or clinical scenarios.

**InterXshift,** the method we propose, leverages these ideas to deliver both a *discovery* and an *estimation* strategy for interaction among continuous exposures. We build upon the insight that if two exposures exceed their additive contribution, they demonstrate synergy (or antagonism, if the joint effect is less than the sum of individual effects). Concretely, we define an *interaction effect* as the difference between (*i*) the expected outcome under a joint stochastic shift of two exposures and (*ii*) the sum of the expected outcomes under separate shifts of each exposure – plus baseline. This echoes classical additive interaction concepts (e.g., RERI) but extends them to continuous multi-exposure contexts in a nonparametric, potential-outcomes framework [1, 2, 17].

### Our Data-Adaptive Procedure to Interaction Discovery and Estimation.

To estimate this interaction parameter in high-dimensional observational data, **InterXshift** uses targeted maximum likelihood estimation (TMLE) [18], deploying flexible machine learning to capture confounding and nonlinearities in both the outcome regression and the exposure density. Because the potential number of pairwise interactions grows quadratically with the count of exposures, we incorporate a *data-adaptive* discovery stage. Specifically, we partition the data into training and validation sets (or folds), rank all pairwise synergies or antagonisms by their estimated departures from additivity in the training portion, and then formally estimate and infer those top interactions using the validation set. This cross-validated approach guards against overfitting, clarifies which pairs matter most, and preserves valid statistical inference even when the parameter being estimated is selected in a data-driven way [19].

Relative to existing mixture-interaction approaches, InterXshift introduces several unique features:

1. **Causal, Model-Agnostic Interaction Definition.** Our parameter is anchored in potential outcomes under stochastic shifts, requiring no specific functional-form assumptions and offering a clear definition of synergy/antagonism in terms of departures from additivity.
2. **Semiparametric Efficiency.** By deriving the efficient influence function for continuous-exposure shifts, we harness TMLE’s strong theoretical guarantees and robust inference properties.
3. **Adaptive Discovery in High Dimensions.** We systematically identify *which* exposure pairs exhibit the strongest synergy or antagonism, reducing the multiple-testing burden common in mixture analysis.
4. **Open-Source Implementation and Validation.** We provide an R package, InterXshift, with comprehensive vignettes and apply it to both synthetic data from the National Institute of Environmental Health Sciences (NIEHS) and real data from the National Health and Nutrition Examination Survey (NHANES) on persistent organic pollutants (POPs) and leukocyte telomere length.

Together, these contributions unite recent advances in causal inference with the practical demands of environmental mixtures research. The remainder of the paper is organized as follows. In Section 2, we outline the stochastic intervention framework and introduce our interaction parameter. Section 3 details the TMLE-based estimation strategy. Section 4.1 explains how we discover key interactions in large exposure sets via cross-validation. In Section 5, we demonstrate performance through simulations, and in Section 6, we showcase real-data applications on synthetic NIEHS mixtures and NHANES POP data. We conclude in Section 8 with a discussion of limitations, possible extensions, and future directions.

## 2 Data and parameter of interest

### 2.1 Overview

At a high level, consider that an analyst has data on a mixture of 15 chemicals (e.g., 15 Persistent Organic Pollutants (POPs)). Our approach first splits the data into a *discovery* sample, used to identify interactions, and an *estimation* sample, used to formally estimate those discovered interactions via targeted maximum likelihood estimation (TMLE). For example, suppose we discover a strong signal of synergy between POP-1 and POP-6 through g-computation [20, 21] on the discovery sample. We then estimate the parameter for that pair in the estimation sample to avoid overfitting bias.

Because our estimand itself (the “top synergy or antagonism”) is data-adaptive, this splitting step is critical for preserving valid statistical inference. Specifically, if the same dataset were used to both discover and estimate interactions, the resulting confidence intervals would be anti-conservative. In practice, this discovery–estimation step can be performed within each fold of a cross-validation scheme, cycling multiple times (e.g., 10 times) to reduce variance. Subsequent sections detail how the parameter is defined and how these interaction-based shifts are constructed.

### 2.2 Notation and framework

We build on the notion of *stochastic interventions* introduced by Díaz and van der Laan [22]. In particular, let

O=(W,A,Y)

be a single observation, where:

– W is a vector of confounders, including both classical covariates (e.g. demographics) *and*, when relevant, other exposures not under direct investigation. For instance, in a multi-exposure setting where the primary focus is on the potential interaction between $A_{1}$ and $A_{2}$, any remaining exposures $A_{3},\ldots ,A_{p}$ that could confound this relationship should be included in W. This practice ensures an appropriate adjustment for correlated influences of secondary exposures on the outcome Y, thereby preserving valid inference for the focal exposures.
– $\text{A}=\left(A_{1},\ldots ,A_{p}\right)$ is the set of main exposures of interest;
– Y is a continuous or binary outcome.

Assume $O_{1},\ldots ,O_{n}$ are i.i.d. from an unknown distribution $P_{0}$. We can factorize

$$P_{0}\left(O\right)=P_{0}\left(Y|\text{A},\text{W}\right)P_{0}\left(\text{A}|\text{W}\right)P_{0}\left(\text{W}\right).$$

Let

$$g_{0}(\text{A}|\text{W})\equiv P_{0}(\text{A}|\text{W})$$

denote the conditional density (or probability mass) of A given W, and let

$${\overset{‾}{Q}}_{0}\left(\text{A},\text{W}\right)\equiv E_{0}\left[Y|\text{A},\text{W}\right].$$

A *shifted* version of $g_{0}$ is defined for a given shift vector δ via

$$g_{\delta }\left(\text{A}|\text{W}\right)=g_{0}\left(\text{A}-\delta |\text{W}\right),$$

leading to a “shifted” density over all variables:

$$p_{\delta }\left(O\right)=p\left(Y|\text{A},\text{W}\right)g_{\delta }\left(\text{A}|\text{W}\right)p\left(\text{W}\right).$$

In many practical scenarios, δ might be a scalar shift (e.g., subtract 1 unit from some pollutant), or a small function of $\left(\text{A},\text{W}\right)$ that ensures *positivity* (i.e., no extreme density ratios). Although we initially treat δ as fixed, we later discuss how it can be scaled adaptively.

#### Remark (Generalized Shifts).

Although we focus primarily on *additive* shifts of the form A↦A−δ, the same stochastic-intervention logic readily accommodates *any bijective* transformation A↦h(A,W). For example, one might apply a *multiplicative* shift A↦aA to uniformly scale exposures by α>0, or a *logistic* re-parameterization to ensure positivity when exposures must remain in a constrained range. In such cases, we replace

$$g_{0}\left(\text{A}-\delta |\text{W}\right)\;\text{by}\;g_{0}\left(h^{-1}\left(\text{A},\text{W}\right)|\text{W}\right)\left|\text{det}\left(Dh^{-1}\left(\text{A},\text{W}\right)\right)\right|,$$

where $\text{det}\left(Dh^{-1}\right)$ denotes the Jacobian determinant of the inverse mapping $h^{-1}$. This recovers the shifted (or otherwise transformed) density under the new exposure law. Thus, one could define a proportionate reduction A↦A×0.8 to reflect a 20% decrease for all individuals, or a more elaborate mapping $\text{A}\mapsto h\left(\text{A},\text{W}\right)$ that depends on individual covariates W. We focus on additive shifts in the main text for conceptual clarity, but the broader framework – constructing $g_{\delta }(\text{A}|\text{W})$ via h(⋅) and its inverse – holds in general.

#### Causal vs. Statistical Parameters.

We can interpret

$$\Psi \left(P\right)=E_{P_{\delta }}\left[Y\right]=\underset{\text{A}}{\int }\underset{\text{W}}{\int }\bar{Q}\left(\text{a},\text{w}\right)g_{\delta }\left(\text{a}|\text{w}\right)p\left(\text{w}\right)\text{d}\text{a}\;\text{d}\text{w}$$

as the mean outcome if a population were subjected to a δ-shift in all components of A. Specifically, we assume A and W admit a strictly positive joint density on the region of interest, so that shifting A by δ remains within its support. Under certain assumptions – namely *ignorability* (Y(a)⊥A∣W) and *positivity* (i.e., $0<g_{\delta }/g_{0}<M$ over the support) – this parameter corresponds to a causal estimand:

$$\Psi^{*}\left(P^{*}\right)=E_{P^{*}}[Y(\text{A}-\delta )].$$

When these assumptions may not hold, Ψ(P) still measures a *statistical* association or partial “what-if” shift, sometimes called a variable-importance parameter [23]. Either way, the shift perspective is appealing in environmental health, where exposures are rarely set to zero but may be reduced or partially controlled.

### 2.3 Interaction target parameter

We now define our *interaction* parameter for two exposures, building on the single-exposure shift described above. Concretely, suppose $\text{A}=\left(A_{i},A_{j},\ldots \right)$, and we wish to see whether $A_{i}$ and $A_{j}$ exhibit synergy (super-additivity) or antagonism (sub-additivity). Let $\delta_{i}$ and $\delta_{j}$ be the shift amounts for each exposure individually; δ then denotes the joint shift of both $A_{i}$ and $A_{j}$. We define:

$$E_{\delta_{i}}[Y]\equiv E_{P_{\delta_{i}}}[Y],\;E_{\delta_{j}}[Y]\equiv E_{P_{\delta_{j}}}[Y],\;E_{\delta }[Y]\equiv E_{P_{\delta }}[Y].$$

Recall that $E_{\delta_{i}}[Y]$ is the mean outcome if we only shift $A_{i}$ by $\delta_{i}$ (keeping $A_{j}$ at its observed levels), and so forth. The expected outcome under a *joint* shift of $A_{i}$ and $A_{j}$ may be additive, supra-additive, or sub-additive. We capture this by the difference from additivity:

$$\Psi_{\text{Interaction}}\left(P\right)=E_{\delta }\left[Y\right]-\left(E_{\delta_{i}}\left[Y\right]+E_{\delta_{j}}\left[Y\right]\right)+E\left[Y\right],$$

where E[Y] is the baseline mean outcome without shifting.

A *positive* $\Psi_{\text{Interaction}}(P)$ implies *super-additivity*, indicating synergy (the combined shift is larger than the sum of parts). A *negative* value implies sub-additivity or antagonism. This mirrors an interaction term in a linear model but is derived here via the stochastic shift framework, making no parametric assumption about the exposure–outcome relationship.

#### Practical Interpretation.

Because shifting continuous exposures requires careful consideration of data support (positivity), we typically use smaller shifts or data-adaptive $\delta_{i}$ that remain in the region where $g_{0}(\text{A}|W)$ is non-negligible. Realistically, an environmental scientist might say “What if we reduce $A_{i}$ by 20% and $A_{j}$ by 10% across the population?” and interpret $\Psi_{\text{Interaction}}(P)$ as how much extra (or reduced) benefit arises from reducing *both* together rather than separately.

#### Three-Way (and Higher-Order) Interactions

Although our primary focus is on two-way synergy or antagonism, one can in principle define a similar *three-way* interaction parameter using stochastic shifts. To motivate this generalization, let

$$E_{\delta_{i}}[Y]=E_{P_{\delta_{i}}}[Y],\;E_{\delta_{j}}[Y]=E_{P_{\delta_{j}}}[Y],\;E_{\delta_{k}}[Y]=E_{P_{\delta_{k}}}[Y],$$

be the expected outcomes under marginal shifts of exposures $A_{i},A_{j}$, and $A_{k}$, respectively. Next, let

$$E_{\delta_{i},\delta_{j}}[Y],\;E_{\delta_{i},\delta_{k}}[Y],\;E_{\delta_{j},\delta_{k}}[Y]$$

denote the means under pairwise shifts, and

$$E_{\delta_{i},\delta_{j},\delta_{k}}[Y]$$

be the mean under a *three-way* shift of $\left(A_{i},A_{j},A_{k}\right)$. Finally, let

$$E[Y]\equiv E_{P}[Y]$$

be the baseline mean outcome (i.e., no exposures are shifted).

A **three-way interaction parameter** can be formulated by comparing the observed triple-shifted mean to all the lower-order shifts and the baseline. Specifically, define:

$$\Psi_{i,j,k}(P)=E_{\delta_{i},\delta_{j},\delta_{k}}[Y]-\left[E_{\delta_{i},\delta_{j}}[Y]+E_{\delta_{i},\delta_{k}}[Y]+E_{\delta_{j},\delta_{k}}[Y]\right]+\left[E_{\delta_{i}}[Y]+E_{\delta_{j}}[Y]+E_{\delta_{k}}[Y]\right]-2E[Y].$$

This can be viewed as a natural extension of *departure from additivity* in the context of stochastic shifts. Concretely,

$$\Psi_{i,j,k}(P)=0$$

would imply that shifting all three exposures simultaneously equals exactly the sum of their single and pairwise contributions (i.e., no three-way interaction). When $\Psi_{i,j,k}(P)>0$, it indicates *super-additivity* (synergy) among the three exposures; when $\Psi_{i,j,k}(P)<0$, it reflects *sub-additivity* (antagonism).

#### Practical Caveats.

Extending from two-way to three-way (or higher-order) interactions quickly expands the number of shifted expectations that must be estimated. This is especially challenging in typical environmental-exposure settings with finite sample sizes and high-dimensional A. Furthermore, the risk of positivity violations and complex correlation patterns grows with each additional exposure. For these reasons, we generally restrict our focus to pairwise interactions, which often hold the greatest practical relevance for regulatory or public-health contexts. However, if the data and sample size permit, one could apply the same semiparametric framework to estimate $\Psi_{i,j,k}(P)$ (and beyond) following the definitions above.

#### Summary.

This section describes the basic setting, from the representation of the data O=(W,A,Y) and the single-exposure stochastic shift, to the *interaction target parameter* that quantifies synergy versus antagonism on an additive outcome scale. In the following sections, we detail how to *estimate* this parameter using targeted maximum likelihood estimation (Section 3), how to *discover* which exposure pairs are most synergistic or antagonistic (Section 4.1), and finally present both simulations (Section 5) and real-world applications (Section 6).

## 3 Estimation and interpretation

In this section, we detail how to estimate the proposed interaction parameter

$$\Psi_{\text{Interaction}}(P)=E_{\delta }[Y]-\left(E_{\delta_{i}}[Y]+E_{\delta_{j}}[Y]\right)+E[Y].$$

We first present the *efficient influence function* (EIF) for shift-based parameters, then describe two approaches for inferring this interaction: (*i*) a separate (“Delta Method”) strategy, and (*ii*) a direct-targeting (single-step) TMLE. We conclude by discussing practical considerations for implementing either approach and how each constructs confidence intervals.

### 3.1 Efficient influence function for shift interventions

#### Basic Idea.

In the targeted maximum likelihood estimation (TMLE) framework, one typically seeks the *efficient influence function* (EIF) of the parameter of interest in order to build an efficient and unbiased estimator. For a single-exposure shift $E_{\delta }[Y]$, Muñoz and van der Laan [17] derived such an EIF. Here, we extend that result to the vector shift $\delta =\left(\delta_{i},\delta_{j}\right)$ for two exposures $A_{i},A_{j}$.

##### Notational Setup.

Let ${\overset{‾}{Q}}_{0}(\text{A},\text{W})\equiv E_{0}[Y|\text{A},\text{W}]$ and $g_{0}(\text{A}|\text{W})\equiv P_{0}(\text{A}|\text{W})$. Shifting A by δ modifies the conditional density to

$$g_{\delta }\left(\text{A}|\text{W}\right)\equiv g_{0}\left(\text{A}-\delta |W\right),$$

leading to

$$E_{\delta }[Y]=\int \int {\overset{‾}{Q}}_{0}\left(\text{a},\text{w}\right)g_{\delta }\left(\text{a}|\text{w}\right)p_{0}\left(\text{w}\right)\text{d}\text{w}\;\text{d}\text{a}.$$

Similarly, define $E_{\delta_{i}}[Y]$ and $E_{\delta_{j}}[Y]$ for shifts of only $A_{i}$ or $A_{j}$.

##### EIF for the Single-Shift Mean.

When shifting A by δ, the EIF of $E_{\delta }[Y]$ is

(1) $$D_{\delta }\left(P_{0}\right)\left(O\right)=H_{\delta }\left(\text{A},\text{W}\right)\left[Y-{\overset{‾}{Q}}_{0}\left(\text{A},\text{W}\right)\right]+{\overset{‾}{Q}}_{0}\left(\text{A}-\delta ,\text{W}\right)-E_{\delta }\left[Y\right].$$

where

$$H_{\delta }\left(\text{A},\text{W}\right)=\frac{g_{0}\left(\text{A}-\delta |\text{W}\right)}{g_{0}\left(\text{A}|\text{W}\right)}.$$

One may think of $H_{\delta }(\text{A},\text{W})$ as the *clever covariate* for the shift-based mean.

#### 3.1.1 Derivation of the clever covariate

Equation (1) highlights that the ratio $\frac{g_{0}(\text{A}-\delta |\text{W})}{g_{0}(\text{A}|\text{W})}$ debiases the naive outcome regression $\overset{‾}{Q}(\text{A},\text{W})$. Conceptually, this ratio arises from the *pathwise derivative* of the shift-based parameter Ψ(P) with respect to g(A∣W). Small perturbations in $g_{0}$ produce changes in Ψ(P) that factor through this ratio, ensuring unbiasedness in the limit. Hence, multiplying $Y-{\overset{‾}{Q}}_{0}(\text{A},\text{W})$ by $H_{\delta }(\text{A},\text{W})$ forces the resulting estimator’s score equation to have mean zero at the truth – a key property for TMLE’s asymptotic efficiency.

#### 3.1.2 EIF for the two-exposure interaction parameter

By linearity, the EIF of

$$\Psi_{\text{Interaction}}(P)=E_{\delta }[Y]-\left(E_{\delta_{i}}[Y]+E_{\delta_{j}}[Y]\right)+E[Y]$$

is simply

$$D\left(P\right)\left(O\right)=D_{\delta }\left(P\right)\left(O\right)-\left[D_{\delta_{i}}\left(P\right)\left(O\right)+D_{\delta_{j}}\left(P\right)\left(O\right)\right],$$

where $D_{\delta },D_{\delta_{i}}$, and $D_{\delta_{j}}$ follow the form in Equation (1) for each shift. Intuitively, the interaction’s EIF subtracts off the individual-shift contributions from the joint shift.

##### Interpretation.

The difference ${\overset{‾}{Q}}_{0}(\text{A}-\delta ,\text{W})-{\overset{‾}{Q}}_{0}(\text{A},\text{W})$ captures how shifting multiple exposures *jointly* affects Y, while

$$H_{\delta }(\text{A},\text{W})\left(Y-{\overset{‾}{Q}}_{0}(\text{A},\text{W})\right)$$

debiases the plug-in outcome regression. Summing this EIF over all observations yields a statistic that (under regularity conditions) has mean zero at the truth, enabling unbiased and efficient estimation of the additive-interaction effect.

### 3.2 Two approaches to estimating ${𝚿}_{\text{Interaction}}$

We now present two complementary TMLE-based strategies for estimating $\Psi_{\text{Interaction}}(P)$. The first estimates each component separately and combines them via the Delta Method; the second directly targets the entire interaction parameter in one step. While both yield valid estimates and asymptotic inference, they differ in complexity and finite-sample trade-offs.

#### 3.2.1 Approach 1: separate estimation (“Delta Method”)

##### Overview.

The “Delta Method” approach calculates ${\hat{E}}_{\delta }[Y],{\hat{E}}_{\delta_{i}}[Y],{\hat{E}}_{\delta_{j}}[Y]$, and $\hat{E}[Y]$ *individually*, each via a standard TMLE for shift parameters [22]. It then combines these four estimates by

$${\hat{\Psi }}_{\text{Interaction}}={\hat{E}}_{\delta }[Y]-\left({\hat{E}}_{\delta_{i}}[Y]+{\hat{E}}_{\delta_{j}}[Y]\right)+\hat{E}[Y].$$

For variance estimation, one applies the usual Delta Method, summing variances and covariances among the four estimated components.

##### Implementation Steps.

1. *TMLE of Each Component*: For each shift $\delta \in \left\{\delta_{i},\delta_{j},\delta \right\}$ plus the baseline E[Y], run a separate shift-based TMLE using Equation (1).
2. *Combine Estimates and Variances*: Form ${\hat{\Psi }}_{\text{Interaction}}$ by linear combination. Approximate the covariance terms with sample covariances of each component’s EIF.
3. *Construct CIs*: Use the estimated variance from the linear combination to get standard errors and confidence intervals.

##### Pros and Cons.

– **Pros:**
  – Straightforward implementation if you already want each marginal shift (${\hat{E}}_{\delta_{i}},{\hat{E}}_{\delta_{j}}$, etc.) individually.
  – Easy to “plug in” new shift magnitudes for any single exposure without redoing the entire interaction calculation.
– **Cons**:
  – May accumulate extra variance from combining multiple separate TMLE estimates, especially in smaller samples.
  – Overlapping nuisance parameters can lead to correlated estimates of the single shifts, inflating covariance terms.

Overall, this method can be *simpler* to implement or interpret when the analyst wants to separately examine each marginal shift. In very large samples, it is often comparable to the direct-targeting approach.

#### 3.2.2 Approach 2: direct targeting (single-step interaction TMLE)

##### Overview.

Rather than estimating each component separately, one can *directly* encode the interaction effect in a *single* TMLE update. This approach constructs a *combined clever covariate* representing $E_{\delta }[Y]-\left(E_{\delta_{i}}[Y]+E_{\delta_{j}}[Y]\right)+E[Y]$ and updates the outcome regression to make the EIF’s empirical mean zero in one pass.

##### Algorithm Steps.

1. *Initial Fits*: Obtain preliminary ${\overset{‾}{Q}}_{n}(\text{A},W)$ and $g_{n}(\text{A}|\text{W})$ from flexible machine learning.
2. *Construct Combined Clever Covariate*:
$$H^{*}(\text{A},\text{W})=\frac{g_{n}(\text{A}-\delta |\text{W})}{g_{n}(\text{A}|\text{W})}-\frac{g_{n}\left(\text{A}-\left(\delta_{i},0\right)|\text{W}\right)}{g_{n}(\text{A}|\text{W})}-\frac{g_{n}\left(\text{A}-\left(0,\delta_{j}\right)|\text{W}\right)}{g_{n}(\text{A}|\text{W})}+1.$$
3. *Update the Outcome Regression*: For a bounded outcome Y, insert $H^{*}(\text{A},W)$ into a logistic (or linear) fluctuation submodel and solve a score equation making the final EIF average zero.
4. *Compute Final Plug-In*: Evaluate the updated regression ${\overset{‾}{Q}}_{n}^{*}$ at $(\text{A}-\delta ),\left(\text{A}-\delta_{i}\right),\left(\text{A}-\delta_{j}\right)$, and A, then combine them to obtain ${\hat{\Psi }}_{\text{Interaction}}$.

##### Pros and Cons.

– **Pros**:
  – Potentially smaller variance in moderate sample sizes, since you target the entire interaction parameter in one shot.
  – Only one submodel fluctuation step is required, which may yield more stable finite-sample performance.
– **Cons**:
  – Implementation is slightly more involved: one must carefully build the “combined” clever covariate.
  – If you later decide to study $E_{\delta_{i}}[Y]$ or $E_{\delta_{j}}[Y]$ alone, you must run a separate TMLE for those single shifts, as they were never individually targeted.

##### Conceptual Trade-Offs.

In smaller samples where synergy or antagonism can be quite strong, direct targeting may reduce finite-sample variance by leveraging a unified submodel. However, if the main interest is separately examining each single shift as well as the interaction, it might be more convenient to use the Delta Method approach. Indeed, many analysts may find it simpler to first estimate $E_{\delta }[Y],E_{\delta_{i}}[Y],E_{\delta_{j}}[Y]$, and E[Y] individually, and only afterward combine them to assess synergy or antagonism as this allows the analyst to interpret the marginal effects as well.

### 3.3 Additional details and practical considerations

#### Bounded vs. Unbounded Y.

If Y is naturally in [0,1] (e.g., a binary outcome), one typically uses a logistic fluctuation in the TMLE update. For continuous unbounded outcomes (e.g., biomarker levels), a linear fluctuation is often simpler:

$${\overset{‾}{Q}}_{n}^{*}\left({\text{A}}_{i},{\text{W}}_{i}\right)={\overset{‾}{Q}}_{n}\left({\text{A}}_{i},{\text{W}}_{i}\right)+\epsilon H^{*}\left({\text{A}}_{i},{\text{W}}_{i}\right).$$

Both approaches solve $\frac{1}{n}\sum_{i}D_{\text{interaction}}(\hat{P})\left(O_{i}\right)=0$. Often, one can rescale Y to [0,1] to allow for a logistic fluctuation even when Y is originally unbounded.

#### Double Robustness and Local Efficiency.

In typical TMLE settings with a single binary treatment, double robustness arises if either the outcome regression $\overset{‾}{Q}$ or the propensity score g is correct. Importantly, this double robustness property extends to our stochastic shift interaction parameter $\Psi_{\text{Interaction}}$. As established in the original work on stochastic interventions by Muñoz and van der Laan [17], consistent estimation is possible if either the outcome regression $\overset{‾}{Q}$ or the conditional density g(A∣W) is correctly specified, although estimating both components well improves efficiency. This double robustness provides an important safeguard against model misspecification in practice. Furthermore, when both components are correctly specified, TMLE remains locally efficient, yielding minimal asymptotic variance among all regular asymptotically linear estimators.

#### Variance and Confidence Intervals.

Regardless of which approach (Delta or Direct Targeting) is used, final inference leverages the empirical variance of the EIF:

$${\hat{\sigma }}^{2}=\frac{1}{n^{2}}\sum_{i=1}^{n}{\left[D_{\text{interaction}}(\hat{P})\left(O_{i}\right)\right]}^{2},$$

where $D_{\text{interaction}}(\hat{P})$ is the *final* influence function under the estimated nuisance parameters. Then, confidence intervals follow as

$${\hat{\Psi }}_{\text{Interaction}}\pm z_{1-\alpha /2}\frac{\hat{\sigma }}{\sqrt{n}}.$$

## 4 Exposure set identification

The estimation of marginal, joint, and interaction shift parameters assumes *a priori* knowledge of important mixture subsets and pairs with statistical interaction evidence. However, in finite samples with many mixture variables A, including all components and possible interactions leads to unacceptable sample variability. We propose a data-adaptive target parameter approach [19] to discover exposure sets and their interaction values through g-computation, then estimate the resulting data-adaptively defined parameters in separate data using TMLE. We employ K-fold cross-validation, splitting the sample data into K approximately equal folds. Let $T_{n,k}\subset 1,\ldots ,n$ denote the k-th training sample indices and $V_{n,k}$ the k-th validation sample indices for k=1,…,K. We use $T_{n,k}$ to identify important set of variables based on the g-computation interaction values and $V_{n,k}$ to estimate our stochastic shift intervention parameters on these exposures.

### 4.1 Exposure set identification

In practice, when investigating multiple exposures $\text{A}=\left(A_{1},\ldots ,A_{p}\right)$, one may wish to identify which pairs (i,j) exhibit the strongest synergy or antagonism under stochastic shifts. Naively enumerating all two-way interactions (there are $\left(\frac{p}{2}\right)$ such pairs) and testing them on the same dataset can result in substantial multiple-testing burdens and inflated type I error if not carefully corrected (e.g., via false-discovery rate (FDR) procedures). Conversely, applying a standard FDR approach might detect fewer signals (less power) if interactions are relatively weak. In this section, we describe a *data-adaptive target-parameter* strategy [19] that splits the data into *discovery* and *estimation* samples, thereby reducing the multiple-test burden by focusing subsequent estimation only on the top candidate interactions.

### 4.2 Cross-validation to discover and estimate interactions

#### Overview of the Two-Stage Procedure.

We propose splitting the full sample into K folds, where $T_{n,k}\subset \{1,\ldots ,n\}$ is the k-th training subset and $V_{n,k}$ is the k-th validation subset (disjoint from $T_{n,k}$). In each fold:

1. **Discovery (Training).** Using only $T_{n,k}$, we fit an algorithm for $\overset{‾}{Q}(\text{A},\text{W})=E[Y|\text{A},\text{W}]$ (via the discrete Super Learner or other flexible ML). We then compute a g-*computation* estimate of our interaction parameter $\Psi_{\text{Interaction,}ij}(P)$ for each pair (i,j), thus obtaining a “rank” of synergy or antagonism.
2. **Estimation (Validation).** The top s synergy pairs and s antagonism pairs (or some user-chosen criterion) form the discovered sets ${𝒮}_{s},{𝒜}_{s}$. We then re-estimate the stochastic shift parameter for these discovered interactions in the *validation* data $V_{n,k}$ using a formal TMLE (Sec. 3).

We repeat the above for k=1,…,K, and combine the fold-specific estimates via cross-validation to get a final inference.

### 4.3 Discovering interactions using training data

#### G-Computation for Ranking.

To identify interactions, we adopt a quick approximate approach in the training fold. Let $\hat{\overset{-}{Q}}$ be the fitted regression for E[Y∣A,W]. For each pair (i,j), define a small shift $\delta_{i},\delta_{j}$ (e.g., subtract 1 unit from each of $A_{i},A_{j}$). We compute

$${\hat{\Psi }}_{\text{Interaction},ij}=\underset{E_{\delta_{i},\delta_{j}}[Y](\text{approx.)}}{\underbrace{\frac{1}{\left|T_{n,k}\right|}\sum_{m\in T_{n,k}}\hat{\overset{-}{Q}}\left(A_{m,i}-\delta_{i},A_{m,j}-\delta_{j},A_{m,-i,j},{\text{W}}_{m}\right)}}-\left({\hat{E}}_{\delta_{i}}[Y]+{\hat{E}}_{\delta_{j}}[Y]\right)+\hat{E}[Y],$$

where each component is evaluated via the same $\hat{\overset{-}{Q}}$ but with the relevant shifts. Comparing ${\hat{\Psi }}_{\text{Interaction,}ij}$ across all (i,j) identifies which pairs appear strongly synergistic (large positive) or antagonistic (large negative).

#### Ranking

Following some rank ordering of ${\hat{\Psi }}_{\text{Interaction,}ij}$ (descending order for synergy, ascending for antagonism), we write

$${𝒮}_{s}=\left\{(i,j):\text{rank}\left[{\hat{\Psi }}_{\text{Interaction,}ij}\right]\leq s\right\},\;{𝒜}_{s}=\left\{(i,j):\text{rank}\left[{\hat{\Psi }}_{\text{Interaction,}ij}\right]\leq s\right\},$$

to indicate the top s pairs. We now treat these ${𝒮}_{s},{𝒜}_{s}$ as our *data-adaptive target parameters* to be estimated in the validation data.

### 4.4 Comparisons to a multiple-testing (FDR) approach

#### Full-Data Testing vs. Discovery-Estimation.

A natural alternative is to test *every* pair (i,j) on the *entire* dataset, applying a multiple-comparisons correction to control the false discovery rate. While feasible, it can be conservative if many interactions are weak. In contrast:

– **Discovery–Estimation** uses a smaller *estimation* set (hence possibly lower nominal power) but avoids heavy multiple-testing penalties by formally testing only the top few discovered pairs.
– **FDR Approach** keeps the full n for each pair’s p-value (arguably more precise if a single pair is of main interest), but must apply e.g. Benjamini–Hochberg corrections across $\left(\frac{p}{2}\right)$ tests, which can reduce power and hamper detection of smaller signals.

In some simulations (not shown), if a large fraction of pairs truly have $\Psi_{\text{Interaction}}\neq 0$, an FDR approach can succeed. But if the number of active interactions is small or the signals are faint, the discovery–estimation approach can be more powerful.

#### Pooling Over Folds for Strength.

An additional advantage of cross-validated discovery is that if the *same* pair (i,j) repeatedly ranks in the top synergy across folds, one effectively “votes” it in, producing an *overall* estimate that uses the entire n. This can recover power lost from splitting. Nonetheless, if results differ drastically across folds (i.e. different top pairs each time), the method indicates that no single pair stands out robustly. Environmental epidemiologists may interpret that as either: (a) no strong, consistent interaction is present, or (b) multiple interactions of similar magnitude exist (leading to instability in ranks). Either way, the cross-validation approach provides a transparent measure of consistency.

#### Interpretation of Inconsistencies Across Folds.

A recurring practical question is how an analyst should interpret results if different folds yield different top-ranked pairs. In our experience, such discrepancies commonly signal at least one of the following scenarios:

1. **No Dominant Interaction.** If no single exposure pair consistently outranks all others in multiple folds, it may suggest there is no strong synergy or antagonism in the dataset; or that the effects are too small relative to sampling variation.
2. **Multiple Interactions of Comparable Magnitude.** When two or more interactions have near-equal effect estimates, cross-validation splits can cause the top ranks to fluctuate.
3. **High Variability or Limited Sample Size.** Environmental data often exhibit strong correlations or small sample sizes, making it difficult for any single pair to dominate consistently.

As a practical step, we advise researchers to track how frequently each pair appears in the top s across folds. If a pair appears in, say, over half the folds, one may consider it a robust candidate for synergy (or antagonism). In contrast, if a pair appears only once or not at all, the signal is likely weak. One may also form the *union* of top interactions from each fold and re-estimate them on a different dataset, providing a final, consolidated inference that can further validate (or refute) whether an interaction is truly important. Another approach is, for a set of ranked synergistic effects, if the same variable pairs are included in the ranks across all the folds, the analyst could in principle report the average rank for the interaction pairs even if there is slight variability in the ranks across the folds.

### 4.5 Estimation of shift interventions for discovered pairs

#### CV-TMLE.

Having identified top synergy/antagonism sets ${𝒮}_{s},{𝒜}_{s}$ in the training data, we estimate the final parameters in the validation data. Concretely, for each fold k:

1. Train nuisance functions ${\overset{‾}{Q}}_{n,k},g_{n,k}$ on $T_{n,k}$.
2. On $V_{n,k}$, compute the TMLE for each discovered pair’s shift-based interaction parameter, controlling for overfitting via cross-validation [19, 24].

This yields a fold-specific interaction estimate and standard error for each pair in ${𝒮}_{s},{𝒜}_{s}$. We then combine across folds to form final estimates and confidence intervals.

### 4.6 Data-adaptive deltas and pooled estimates

#### Positivity Constraints and Adaptive Shifts.

Choosing a fixed shift magnitude δ can lead to extreme density ratios $\frac{g_{n}(\text{A}-\delta |\text{W})}{g_{n}(\text{A}|\text{W})}$ if the shifted values fall outside the natural support. In particular, if δ is too large, then for at least one observation i, the ratio

$$H_{\delta }\left({\text{A}}_{i},{\text{W}}_{i}\right)=\frac{g_{n}\left({\text{A}}_{i}-\delta |{\text{W}}_{i}\right)}{g_{n}\left({\text{A}}_{i}|{\text{W}}_{i}\right)}$$

could “blow up” beyond a practical threshold. To avoid this violation of positivity, one may *adaptively shrink* δ within each fold. Concretely, one can start with a candidate δ and evaluate $H_{\delta }\left({\text{A}}_{i},{\text{W}}_{i}\right)$ for all observations in the training fold. If any ratio exceeds a user-defined ceiling (e.g. λ=50), δ is reduced (e.g. by stepping down on a grid or via bisection) until ${\text{max}}_{i}H_{\delta }\left({\text{A}}_{i},{\text{W}}_{i}\right)<\lambda$. Because ratios less than 1 do not typically pose the same risk of positivity violations (they reflect moving an observation into a denser region), we do not impose a symmetric lower bound. In practice, our software automates this iterative procedure; users need only specify the initial δ and the threshold λ.

#### Averaging Shifts Across Folds.

In the case of adaptive shifts, because δ may differ across folds (depending on each fold’s empirical distribution), we then “average” the chosen shifts into a single $\overset{‾}{\delta }$. Specifically, if $\delta_{k}$ is the largest feasible shift in fold k, we define $\overset{‾}{\delta }=\frac{1}{K}\sum_{k=1}^{K}\delta_{k}$. We pair this average shift with the previously estimated nuisance components to produce a single, final shift-based estimate. That is, we do a pooled TMLE update with the nuisance parameters (that may be based on different shifts) and pair this estiamte with the average shift across the folds. While $\overset{‾}{\delta }$ is more complicated to interpret than a pre-fixed shift, it helps prevent positivity issues in at least some folds and thus provides an *average feasible* shift across the population. In many analyses, however, one might simply choose a modest fixed δ well within the data’s support and forego adaptivity entirely – especially if no substantial positivity concerns are apparent.

### 4.7 Exposure density estimation

Estimating the joint density of A given covariates W is central to our stochastic-shift parameter. In particular, we require $g_{n}(\text{A}|W)$ – the conditional density (or probability mass) of the exposures – both to construct the clever covariates for TMLE and to implement the shifts $g_{\delta }(\text{A}|W)$. Although this step may appear straightforward, it can pose significant challenges in practice, especially in high-dimensional data or when A includes heterogeneous continuous exposures. This section provides an overview of the two main strategies currently supported by our approach and software, while also highlighting alternative ratio-estimation methods from recent literature.

#### Direct Modeling of the Conditional Density.

A natural approach is to model g(A∣W) directly using machine-learning regressors tailored to density estimation. For instance, one can employ parametric or semiparametric density estimators (e.g., mixture models) or nonparametric machine-learners (e.g., random forests or gradient boosting) that predict the density of A given W. We then form the ratio

$$\frac{g_{n}(\text{A}-\delta |\text{W})}{g_{n}(\text{A}|\text{W})}$$

as needed to define the clever covariate for the shift. While conceptually straightforward, direct density modeling can be difficult in high dimensions or with multi-modal, heavily skewed exposure distributions. It also demands careful hyperparameter tuning to avoid instability in ratio estimates. In our software, Super Learner [25] is used to select the best density estimator from a set of candidates based on cross-validated loss; however, machine learning density estimators are substantially lacking compared to classification counterparts.

#### Classification-Based Reparameterization.

An alternative, and often more robust, strategy recasts density-ratio estimation as a binary classification problem in an *augmented* dataset [26,27]. We duplicate each observation $\left({\text{W}}_{i},{\text{A}}_{i}\right)$ and label one copy as “shifted” (ξ=1), meaning its exposure vector is replaced by ${\text{A}}_{i}-\delta$, and the other copy as “unshifted” (ξ=0). A flexible classifier (e.g., a Super Learner ensemble) then predicts P(ξ=1∣W,A). From Bayes’ rule, the probability ratio

$$\frac{P(\xi =1|\text{W},\text{A})}{P(\xi =0|\text{W},\text{A})}$$

directly encodes $\frac{g(\text{A}-\delta |\text{W})}{g(\text{A}|\text{W})}$, yielding the necessary clever-covariate component. This classification-based approach often proves more numerically stable than direct density modeling, thanks to the maturity of binary classification algorithms and regularizers for high-dimensional data.

#### Implementation in InterXshift.

Our software package supports *both* direct density modeling and classification-based reparameterization. In either case, users can leverage the Super Learner [25] framework to combine multiple candidate learners (e.g., generalized linear models, random forests, gradient boosting) adaptively. We default to the classification-based approach in high-dimensional settings for its computational stability, but direct density modeling may be appropriate when prior knowledge suggests a particular parametric or semiparametric form for g(A∣W).

#### Advanced Ratio-Estimation Alternatives.

Recent methodological developments offer additional tools that could be readily integrated into our framework. For instance,

– *Neural-network-based importance sampling* methods [28] learn flexible parametric forms of the exposure distribution that can be advantageous for extremely high-dimensional A, or for imposing specific structural assumptions (e.g., normalizing-flow models).
– *Energy balancing* [29] is another emerging strategy for density-ratio estimation that aims to match the distributions of shifted and unshifted samples by minimizing an energy distance. This approach can help address positivity violations or regions of low overlap in the data, potentially improving finite-sample stability.

While we have not yet implemented these novel ratio-estimation strategies in InterXshift, their inclusion would be conceptually straightforward. We view such innovations as a promising direction for future research, particularly in scenarios where classical machine-learning or classification-based approaches struggle with very large or complex exposure spaces.

Overall, the density-estimation step must not be viewed as trivial. Choosing among direct modeling, classification-based reparameterization, or more recent specialized approaches can profoundly influence both the numerical stability of the algorithm and the accuracy of the final stochastic-shift estimates.

## 5 Simulations

We conducted a simulation study to assess the performance of InterXshift under a range of synergy and antagonism strengths, with particular focus on (i) identifying the correct interacting exposures and (ii) accurately estimating interaction parameters. The scenarios we consider approximate small, moderate, and large interaction magnitudes, reflecting effect sizes plausible in environmental exposure settings.

### 5.1 Data-generating mechanism (DGM)

We extend the basic framework described in Section 2 to include two explicit interaction terms among six exposures, $\left(A_{1},A_{2}\right)$ and $\left(A_{5},A_{6}\right)$. Specifically, we generate confounders

$$\text{W}=\left(W_{1},\ldots ,W_{4}\right)\sim 𝒩(0,𝚺),$$

where 𝚺 encodes moderate pairwise correlations (e.g., 0.2–0.4). Next, for each observation, we sample six continuous exposures $\text{A}=\left(A_{1},\ldots ,A_{6}\right)$ via gamma, beta, or truncated-normal distributions. These distributions are chosen to mimic features commonly seen in environmental exposures, such as right-skew (lognormal-like) or bounded support (beta).

We then define the outcome Y as

$$Y=1.0+0.3W_{1}+0.2W_{2}+0.3A_{1}+0.2A_{2}-0.3A_{3}+\text{Synergy}\;\text{Strength}\left(A_{1}\times A_{2}\right)+\text{Antagonism}\;\text{Strength}\left(A_{5}\times A_{6}\right)+0.2\left(A_{4}^{2}\right)+\varepsilon ,$$

with ε~𝒩(0,1). Here, W confounds A and Y because W simultaneously affects the exposures and has a direct effect on Y. By shifting A while adjusting for W, we emulate realistic observational data and explore how well InterXshift uncovers synergy in this continuous exposure setting. Hence, while we included both synergy and antagonism terms in the data-generating mechanism, we focus primarily on detecting synergy in these simulations for clarity and succinctness; there is little reason to believe synergy is either more or less challenging to estimate than antagonism, and our aim here is to examine whether weak synergy signals remain detectable under realistic confounding and correlated exposures with additional interactions.

### 5.2 Simulation scenarios and sample sizes

To capture a spectrum of interaction intensities, we vary the terms:

– **SynergyStrength** ∈ {0.25, 0.40, 0.60} (small, moderate, large positive interaction between $A_{1}$ and $A_{2}$);
– **AntagonismStrength** ∈ {−0.20, −0.45, −0.70} (small, moderate, large negative interaction between $A_{5}$ and $A_{6}$).

These ranges were selected to reflect additive-scale interaction sizes typical of modest (0.20–0.40) or more pronounced (0.50–0.70) synergistic or antagonistic effects found in certain environmental exposures.

We consider sample sizes n∈{200,500,1,000,2,000}. Smaller n settings allow us to assess performance when data are relatively limited, a common situation in environmental epidemiology. Larger n illustrates whether $\sqrt{n}$-rate consistency emerges as theory predicts.

### 5.3 True values of synergy

To evaluate estimation accuracy, we compare each estimated interaction to its “true” value, computed via a large sample (e.g., n=100,000) from the same DGM. Concretely, for our interaction parameter, we define

$$\Psi_{\text{interaction}}=E_{P_{\delta_{1},\delta_{2}}}\left[Y\right]-\left(E_{P_{\delta_{1}}}\left[Y\right]+E_{P_{\delta_{2}}}\left[Y\right]\right)+E_{P}\left[Y\right],$$

where $\delta_{1}$ and $\delta_{2}$ shift $A_{1}$ and $A_{2}$ by 0.5 unit each, chosen to lie within the typical range of $A_{1}$ and $A_{2}$. We approximate each expectation $E_{P_{\delta }}[Y]$ by sampling from the DGM at very large n, thus obtaining stable reference values for comparison.

### 5.4 Implementation

1. **Data Generation:** Draw n i.i.d. observations $\left({\text{W}}_{i},{\text{A}}_{i},Y_{i}\right)$ from the above DGM.
2. **Cross-Validation for Discovery–Estimation:**
  – **Discovery Stage:** Fit flexible ML models for $\overset{‾}{Q}(\text{A},\text{W})$ and g(A∣W) on the training fold. For each exposure pair, compute a g-computation approximation of synergy. Rank pairs by positive (synergy) or negative (antagonism) values.
  – **Estimation Stage:** For the top-ranked synergy pair and the top-ranked antagonism pair, estimate the final interaction parameter $\Psi_{\text{Interaction}}$ via targeted maximum likelihood estimation (TMLE), using only the validation fold.
3. **Repetition and Summary:** Repeat for N replicates (e.g., 100) under each scenario and sample size. Pool the estimates across folds to obtain $\hat{\Psi }$ and its corresponding confidence interval in each replicate.
4. **Comparison to Truth:** Compare $\hat{\Psi }$ to $\Psi_{\text{true}}$ (Section 5.3) to quantify bias and coverage.

### 5.5 Performance metrics

We summarize each simulation’s output over N replicates:

– **Mean Squared Error (MSE):**
$$\text{MSE}={\text{Bias}}^{2}+\text{Var}\left(\hat{\Psi }\right).$$
– **95% Coverage:** Fraction of replicates whose 95% CIs contain $\Psi_{\text{true}}$.
– **Scaled Bias:** $|\hat{\text{Bias}}|\times \sqrt{n}$, to check near-$\sqrt{n}$ consistency.

### 5.6 Synergy results and key findings

**Figure 1:**
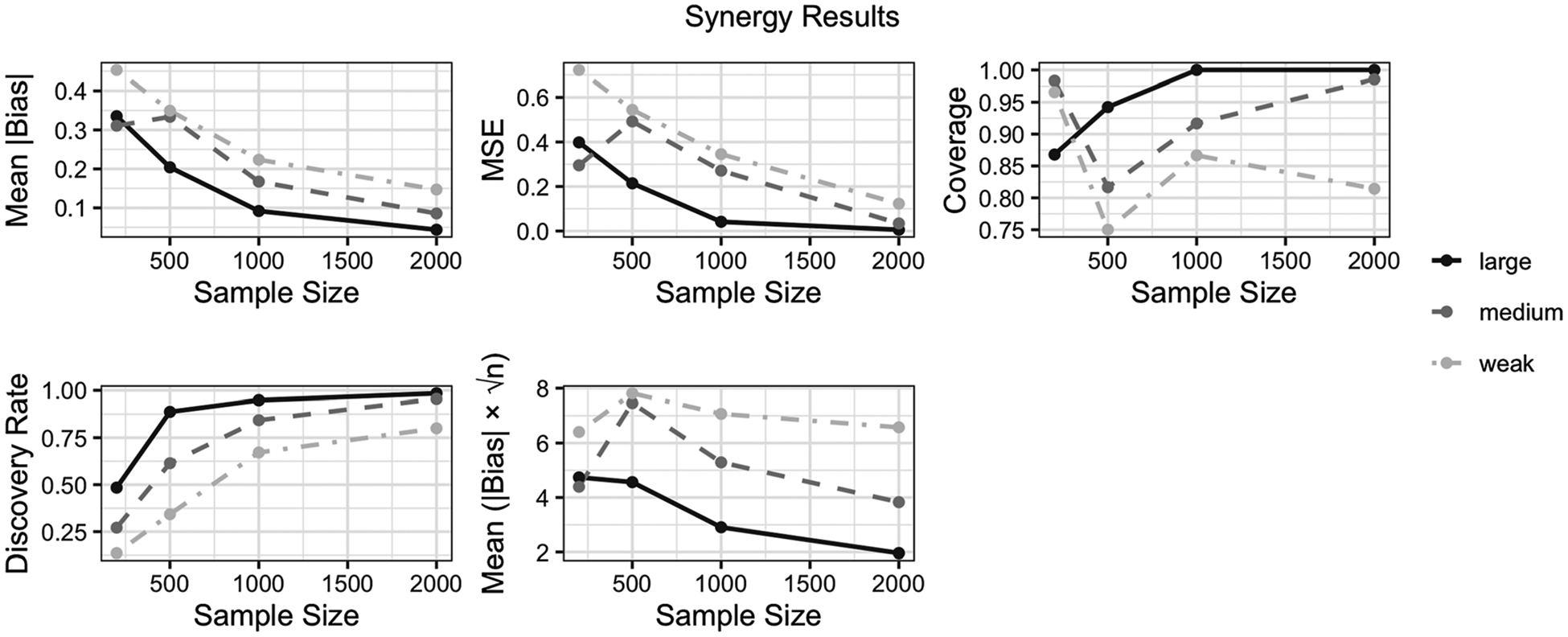
Synergy simulation results. Five panels present key performance metrics of InterXshift for detecting and estimating synergy in the mixture of six exposures. From left to right, the plots display (i) mean absolute bias, (ii) mean squared error (MSE), (iii) coverage of the 95% confidence intervals, (iv) discovery rate of the correct synergy pair $\left(A_{1},A_{2}\right)$, and (v) scaled bias $|\text{Bias}|\times \sqrt{n}$. Sample sizes range from n=200 to n=2,000, and synergy strengths are classified as weak (0.25), medium (0.40), or large (0.60). As n increases, both bias and MSE decline substantially, coverage approaches 95%, and scaled bias remains stable or decreases, indicating $\sqrt{n}$-rate convergence of the targeted maximum likelihood estimator. Overall, InterXshift demonstrates strong performance in detecting and accurately quantifying additive-scale synergy in confounded, continuous-exposure data.

We evaluated the performance of InterXshift in detecting and estimating a positive interaction (synergy) between exposures $\left(A_{1},A_{2}\right)$ across three effect magnitudes: weak (0.25), moderate (0.40), and large (0.60). Figure 1 displays the method’s behavior for a range of sample sizes (n=200,500,1,000,2,000) in terms of mean absolute bias, mean squared error (MSE), coverage, discovery rate, and scaled bias $|\text{Bias}|\times \sqrt{n}$. Below we highlight the principal findings:

#### (1) Accurate Discovery of the Synergistic Pair.

Even with relatively small samples (n=500), InterXshift correctly ranked $\left(A_{1},A_{2}\right)$ among the top synergy pairs in most replicates when the true synergy was moderate or large. Occasional misses arose under the weakest synergy (0.25) combined with small n, illustrating that detecting subtle interactions can require additional data. Nonetheless, by n=1,000 and above, the discovery rate consistently exceeded 75% for weak synergy and approached or exceeded 90% for moderate and large synergy.

#### (2) Reduced Bias and MSE With Increased Sample Size.

As shown in Figure 1, the mean absolute bias and MSE decreased markedly with n. At n≤500, absolute bias ranged from roughly 0.2 to 0.35 across different synergy strengths, whereas at n=1,000 or 2,000, both bias and MSE became notably smaller (often under 0.10 bias for moderate/large synergy). These patterns reflect the benefits of having more observations for the targeted maximum likelihood estimation (TMLE) procedure to learn both outcome and exposure-density relationships.

#### (3) Confidence Interval Coverage Approaching 95%.

Coverage for moderate and large synergy converged toward the nominal 95% level by n=1,000, typically reaching or exceeding 90% at n=500. Even for weak synergy, coverage climbed steadily from around 75–80% at n=200 to near 90% at n=1,000. These findings support the theoretical robustness of TMLE in finite samples, provided that key positivity conditions are not seriously violated.

#### (4) Stable or Decreasing Scaled Bias.

A key indicator of $\sqrt{n}$-rate convergence is whether $|\text{Bias}|\times \sqrt{n}$ remains roughly constant or declines with increasing n. Consistent with TMLE theory, we observed that scaled bias either plateaued or decreased across larger sample sizes, suggesting that InterXshift can achieve efficient asymptotic performance under flexible machine-learning estimation of nuisance functions.

##### Overall Implications for Environmental Data.

These results underscore InterXshift’s practical feasibility in typical environmental-mixture studies. Even when exposures are moderately correlated and confounded by covariates, the method can reliably detect and quantify an additive-scale synergy – particularly as sample sizes surpass a few hundred observations. While very weak interactions and limited data may hamper detection, the consistent reduction in bias and growth in coverage at higher n reflect the benefits of a data-adaptive discovery-and-estimation framework. Thus, InterXshift offers a robust solution for isolating key synergies in multi-exposure settings and guiding follow-up toxicological or epidemiologic investigations.

## 6 Applications

### 6.1 Analysis of NIEHS synthetic mixtures data

The NIEHS synthetic mixtures data is a commonly used data set to evaluate the performance of statistical methods for mixtures. These synthetic data can be considered the results of a prospective cohort study, where the outcome cannot cause exposures, and correlations between exposure variables can be thought of as caused by common sources or modes of exposure. The variable W can be assumed to be a potential confounder and not a collider. The data set has 7 exposures $\left(A_{1}-A_{7}\right)$ with a complex dependency structure based on endocrine disruption. Two groups of exposure ($A_{1},A_{2},A_{3}$ and $A_{5},A_{6}$) lead to high correlations within each group. $A_{1},A_{2},A_{7}$ positively contribute to the outcome, $A_{4},A_{5}$ have negative contributions, while $A_{3}$ and $A_{6}$ have no impact on the outcome. Rejecting $A_{3}$ and $A_{6}$ is difficult due to their correlations with the members of the cluster group. This correlation and effects structure is biologically plausible, as different congeners of a group of compounds may be highly correlated but have different biological effects. Exposures have various agonistic and antagonistic interactions, a breakdown of the variables sets and their relationships are: $A_{1},A_{2}$ both increase Y by concetration addition, $A_{1}-A_{4},A_{2}-A_{4},A_{1}-A_{4},A_{2}-A_{4}$ competetive antagonism, $A_{1}-A_{7},A_{2}-A_{7}$ supra-additive or synergistic, $A_{4},A_{5}$ both addivively decrease Y, $A_{4}-A_{7},A_{5}-A_{7}$ unusual antagonism. Synthetic data and the key for dataset 1 are available on GitHub. This resource shows the interactions and marginal dose relationships built into the data. Given these toxicological interactions, we expect these sets of variables to be determined in InterXshift. For example, we might expect a positive counterfactual result for A1, A2, A7 and negative results for A4 and A5. Table 1 shows the interactions built into this data.

Likewise, in the case for antagonistic relationships such as A1–A5 (third row in 1), we would expect a joint shift to come closer to the null, since A5 antagonizes the positive effects of A1. For A1 and A2, we would expect the joint shift to be close to the sum of individual shifts (not much interaction), but for A1 and A7 we expect to estimate more than an additive effect (some interaction). The NIEHS data set has 500 observations and 9 variables. W is a binary confounder. Based on this table, we can gauge InterXshift’s performance by determining if the correct synergistic and antagonistic relationship are found and if the correct variables are rejected.

We apply InterXshift to these NIEHS synthetic data using a three-fold CV (smaller CV to save space here in k-fold result tables). We use the default libraries of learners for the estimation of ξ and Q. We parallelize over the cross-validation to test computational run-time on a newer personal machine an analyst might be using. We use a delta of 1 for all the exposures in the mixture. We present the pooled TMLE results and the k-fold specific results.

### 6.2 NIEHS synthetic data results

InterXshift accurately rejects exposures $A_{3},A_{6}$, finding the correct top positive marginal effects of $A_{1}$ in all the folds and the top inverse association $\left(A_{5}\right)$ in all the folds. Likewise, we found the correct synergistic results built into the DGP for $A_{1}-A_{7}$ and $A_{2}-A_{7}$. In all the folds, $A_{5}-A_{7}$ was found to have the strongest antagonistic relationship, which is also true based on the DGP key provided from the NIEHS.

Table 2 presents the rank 1 positive marginal results for the NIEHS data. The first three rows display fold-specific results, with Ψ representing the expected change in the outcome under the exposure shift compared to the average observed outcome. Here, $A_{1}$ is identified as the top positively associated mixture variable across all folds. The pooled TMLE column provides the aggregate results, showing an average Ψ of 16.31 with a standard error of 0.37 and a 95% confidence interval of 15.59–17.04. These results underscore the consistent positive association of $A_{1}$ with the outcome across different data folds.

Table 3 presents the rank 1 inverse marginal results for the NIEHS data. The first three rows show fold-specific results, with Ψ indicating the expected change in the outcome under the exposure shift compared to the average observed outcome. Here, A5 is identified as the top negatively associated mixture variable across all folds. The pooled TMLE column provides the aggregate results, demonstrating that a 1-unit increase in A5 leads to an average decrease of −3.67 in endocrine disruption, with a standard error of 0.50 and a 95% confidence interval of −4.65 to −2.70. These results highlight the consistent negative association of A5 with the outcome across different data folds.

Table 4 presents the rank 1 synergistic interaction results for the NIEHS data. The table shows fold-specific results, with Ψ indicating the expected change in the outcome under a 1-unit shift in the specified exposure(s). Here, we observe that A1 and A7 are identified as the top synergistic relationship in two folds, while A2 and A7 are identified in the remaining fold. The joint shift for A1 and A7 and for A2 and A7 reveals significant synergistic interactions built into the data-generating process. The interaction effects reflect the combined effect of shifting both exposures compared to the sum of their individual shifts, indicating strong synergy where the Psi under interaction is positive.

Table 5 presents the pooled TMLE estimates for the rank 1 synergistic relationships in the NIEHS data. The first two rows show the average individual effects of the variables involved in the top synergistic relationships, identified as Var 1 and Var 2, here because Var 1 changed across the folds it is the average across A1 and A2 and Var 2 corresponds to A7. The third row represents the joint effect of shifting both exposures simultaneously, while the fourth row shows the average synergistic interaction effect, which is significantly greater than the sum of individual shifts. This pooled estimate reflects the average effect across the identified top synergistic relationships.

Table 6 presents the k-fold specific antagonistic rank 1 results for the NIEHS data. The Ψ column represents the expected change in the outcome under a 1-unit shift in the specified exposures. For the first fold, variable A5 shows a negative shift of −3.97, indicating a strong inverse association. Similarly, variable A7 shows a positive shift of 3.00. The combined shift of A5 and A7 results in an estimated change of −1.76, while the interaction term, which compares the joint shift to the two marginal shifts, shows a negative shift of −0.78. These patterns are consistent across all three folds, demonstrating significant evidence for antagonistic interactions between these variables.

#### 6.2.1 Comparison to existing methods

Quantile g-computation [5], prevalent in environmental epidemiology for mixture analysis, estimates the effects of uniformly increasing exposures by one quantile, based on linear model assumptions. This method quantizes mixture components, summing the linear model’s coefficients to form a summary measure (Ψ) for joint impact assessment. However, it inherently assumes additive, monotonic exposure-response relationships, overlooking complex, potentially nonlinear interactions typical in mixtures, like in endocrine disrupting compounds. Consequently, this method might not accurately capture the nuanced dynamics of mixed exposures, especially when interactions vary with other variable levels.

We run quantile g-computation on the NIEHS data using 4 quantiles with no interactions to investigate results using this model. The size of the scaled effect (positive direction, sum of positive coefficients) was 6.28 and included $A_{1},A_{2},A_{3},A_{7}$ and the scaled effect size (negative direction, sum of negative coefficients) was −3.68 and included $A_{4},A_{5},A_{6}$. Compared to NIEHS ground truth, $A_{3},A_{6}$ are incorrectly included in these estimates. However, the positive and negative associations for the other variables are correct. Next, because we expect interactions to exist in the mixture data, we would like to assess for them but the question is which interaction terms to include? Our best guess is to include interaction terms for all exposures. We do this and show results in Table 7.

In Table 7 $\Psi_{1}$ is the summary measure for the main effects and $\Psi_{2}$ for interactions. As can be seen, when including all interactions, neither of the estimates are significant. Of course, this is to be expected given the number of parameters in the model and the sample size n=500. However, moving forward with interaction assessment is difficult; if we were to assess for all 2-way interaction of 7 exposures, the number of sets is 21 and with 3-way interactions is 35. We would have to run these many models and then correct for multiple testing. Hopefully, this example shows why mixtures are inherently a data-adaptive problem and why popular methods such as this, although succinct and interpretable, fall short even in a simple synthetic data set.

### 6.3 Comparison with a group lasso-based approach (glinternet)

Although quantile g-computation is a commonly used screening tool for mixtures, it was never designed to systematically isolate pairwise interactions. We therefore also compared InterXshift with a penalized-regression strategy that *does* attempt automatic interaction detection in high dimensions. Specifically, we employed the glinternet algorithm [30], which fits a generalized linear model with hierarchical pairwise interactions using an $\ell_{1}$-type group-lasso penalty. By penalizing the inclusion of main and interaction terms together, glinternet can parsimoniously select or discard interaction effects without manual enumeration of all pairwise products – a feature appealing in high-dimensional mixture settings [7, 9].

#### Implementation on the NIEHS Synthetic Data.

We applied glinternet (with a Gaussian family) to the NIEHS synthetic dataset described in Section 6, using five-fold cross-validation to choose the optimal penalty level. Following standard practice, we adopted the “one-standard-error” rule, favoring a more parsimonious solution whenever multiple penalties produced near-equal cross-validation errors. Table 1 (Section 6) lists the ground-truth interactions built into the synthetic data – e.g., $\left(A_{1},A_{7}\right)$ and $\left(A_{2},A_{7}\right)$ exhibit supra-additive synergy, whereas $\left(A_{4},A_{5}\right)$ and $\left(A_{5},A_{7}\right)$ exhibit antagonism, and $\left(A_{3},A_{6}\right)$ are null exposures.

#### Key Findings and Comparison to Ground Truth.

Table 8 shows the main and interaction terms selected by glinternet, along with their estimated coefficients under the cross-validated model. On the positive side, glinternet captured several true interactions, including $\left(A_{1},A_{7}\right),\left(A_{2},A_{7}\right)$, and $\left(A_{5},A_{7}\right)$ – all of which appear in the synthetic data dictionary. However, several discrepancies arose:

##### (1) Negative Coefficients for Pairs That Are Actually Synergistic.

For instance, glinternet discovered $\left(A_{1},A_{2}\right)$ but assigned a *negative* interaction coefficient. Biologically, $A_{1}$ and $A_{2}$ are nearly toxic equivalents that *increase* Y in an additive or slightly super-additive way. In a linear model, if $A_{1}$ and $A_{2}$ are strongly correlated and both positively correlated with Y, this can paradoxically yield a negative *interaction* term, reflecting how the model partitions the linear predictor among correlated covariates. Thus, the sign of a glinternet interaction coefficient does *not* necessarily indicate super- or sub-additivity in the usual epidemiologic sense.

##### (2) Spurious Interactions Involving Null Exposures.

glinternet also selected terms involving $\left(A_{3},A_{6}\right)$ – exposures that the NIEHS synthetic key designates as inactive. This likely reflects the model’s attempt to fit residual correlation patterns in Y. Because glinternet estimates a linear projection of Y onto main and product terms, even exposures with zero true additive effect can appear if they help reduce squared error by capturing correlated structures. By contrast, InterXshift – which quantifies synergy via *departures from additivity under hypothetical exposure shifts* – did not single out $A_{3}$ or $A_{6}$ as having strong interactions.

##### (3) Linear vs. Shift-Based Interpretations.

By design, glinternet fits a penalized linear model, so an “interaction” is merely a coefficient for $A_{i}\times A_{j}$ on the linear predictor scale. InterXshift, on the other hand, defines synergy or antagonism via the difference

$$E_{\delta }[Y]-\left(E_{\delta_{i}}[Y]+E_{\delta_{j}}[Y]\right)+E[Y],$$

i.e., an explicit causal contrast comparing a joint shift to separate individual shifts. Consequently, a large positive coefficient in glinternet *can* correspond to sub-additive behavior for the actual outcome, or vice versa.

#### Implications for Environmental Mixtures.

Our findings underscore that glinternet can help *screen* for interaction candidates in large exposure sets. Its selection mechanism is relatively fast and requires minimal manual specification of product terms. However, the interpretability of glinternet’s interaction coefficients is limited when the scientific question centers on truly additive synergy (or antagonism). In particular:

– A negative penalized coefficient for $\left(A_{i},A_{j}\right)$ may not mean that $A_{i}$ and $A_{j}$ are antagonistic in an epidemiologic sense, but rather that, given the partial correlations, the linear model “subtracts” from one slope when the other exposure is present.
– Even null exposures can be selected if they slightly improve predictive fit – leading to additional product terms that do not align with the synthetic data’s known synergy/antagonism structure.

By contrast, InterXshift relies on a direct, causal definition of “departure from additivity” via population-wide shift interventions. This often yields more transparent estimates for public-health and policy discussions – e.g., “Would reducing chemical $A_{1}$ by 1 unit and $A_{7}$ by 1 unit together yield more or less benefit than reducing each alone?” Nonetheless, penalized methods like glinternet offer a pragmatic exploratory tool, and can complement our shift-based approach by quickly highlighting which pairs warrant deeper investigation.

### 6.4 Methods applied to NHANES dataset

The National Health and Nutrition Examination Survey (NHANES) 2001–2002 cycle serves as the foundation of our analysis. This data source, known for its credibility in the public health domain, boasts interviews with 11,039 individuals. Of this subset, 4,260 provided blood samples and willingly consented to DNA analysis. The data set used aligns with that of Mitro et al. [31] focusing on the correlation between persistent exposure to organic pollutants (POPs), specifically those binding to the aryl hydrocarbon receptor (AhR) and extended leukocyte telomere length (LTL). However, this subset was further refined to ensure complete exposure data, yielding 1,007 participants for our analysis, compared to 1,003 in the initial study by Mitro et al. In alignment with protocols detailed by Mitro et al. [31], exposure was quantified, focusing on 18 congeners. These include 8 non-dioxin-like PBCs, 2 non-ortho PCBs, 1 mono-ortho PCB, 4 Dioxins, and 4 Furans. All congeners underwent lipid serum adjustments using an enzymatic summation methodology. The telomere length, was analyzed using the quantitative polymerase chain reaction (qPCR) methodology [31]. This technique measures the T/S ratio by comparing the length of the telomere to a standardized reference DNA. To enhance accuracy, samples underwent triple assays in duplicate, generating an average from six data points. The CDC conducted a blinded quality control assessment. Our modeling accounted for several covariates, including demographic factors such as age, sex, race/ethnicity and level of education, as well as health indicators such as BMI, serum cotinine, and blood cell distribution and count. Categorizations for race/ethnicity, education, and BMI are consistent with previous studies. Furthermore, this comprehensive dataset is integrated into the InterXshift package for replicable analysis.

**Figure 2:**
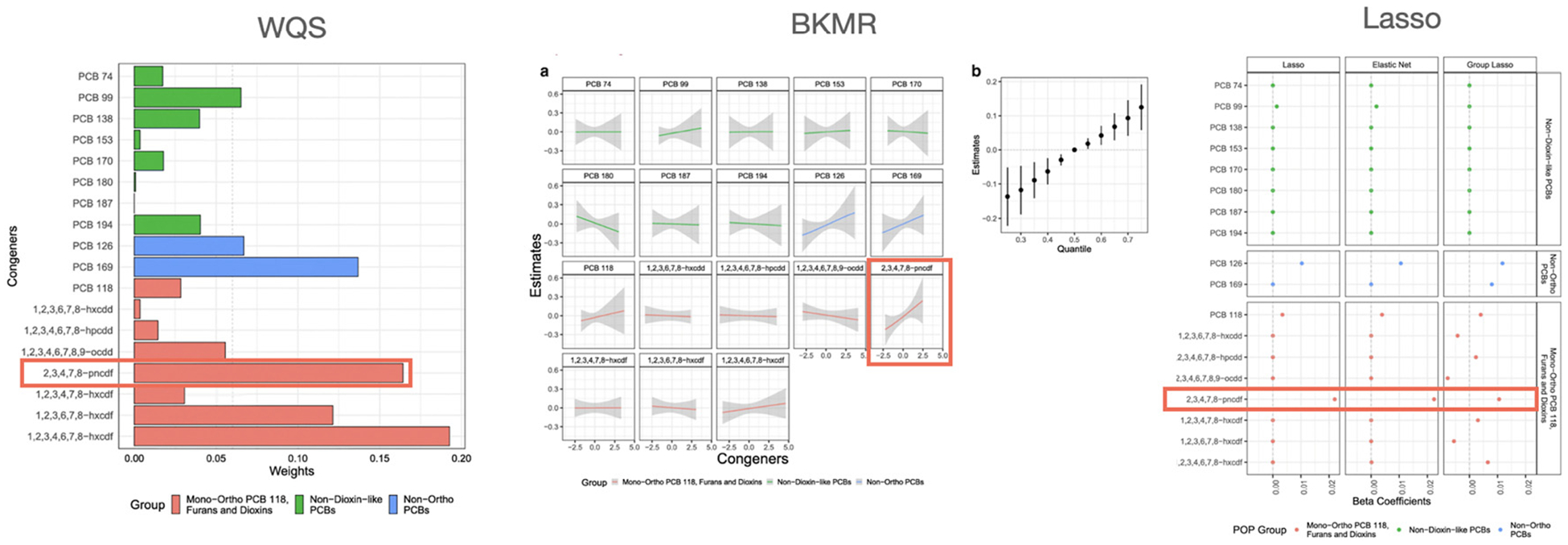
Mixture methods results from Gibson et al.

Gibson et al. [6] reanalyzed this same data using more contemporary statistical methods. They found that clustering methods identified high, medium, and low POP exposure groups with longer log-LTL observed in the high exposure group. Principal component analysis (PCA) and exploratory factor analysis (EFA) revealed positive associations between overall POP exposure and specific POPs with log-LTL. Penalized regression methods identified three congeners (PCB 126, PCB 118, and furan 2,3,4,7,8-pncdf) as potentially toxic agents. WQS identified six POPs (furans 1,2,3,4,6,7,8-hxcdf, 2,3,4,7,8-pncdf, and 1,2,3,6,7,8-hxcdf, and PCBs 99, 126, 169) as potentially toxic agents with a positive overall effect of the POP mixture. BKMR found a positive linear association with furan 2,3,4,7,8-pncdf, suggestive evidence of linear associations with PCBs 126 and 169, a positive overall effect of the mixture, but no interactions among congeners. These results (in the supervised methods) controlled for the same covariates. Figure 2 shows the results for WQS, BKMR and Lasso regression from their paper. We highlight furan 2,3,4,7,8-pncdf as the chemical which shows consistent positive association with LTL across the methods. As such, we might expect that this chemical would consistently be found as the top positive marginal impact based on our target parameter across all the folds.

We configured our methodology using default learners and employed a 5-fold CV. Given the data range, a δ value of half a standard deviation was used for all exposures, denoting a focus on counterfactual changes in telomere length for an increase in half a standard deviation across exposures that predict the telomere length depending on the scale of exposure.

### 6.5 NHANES furan results

Generally, when applying our InterXshift method to identify the top positive and negative marginal effects; and the top synergistic and antagonistic interactions, we do not find consistent estimates for these parameters. Table 9 shows the estimates of the top positive association between the folds. Although we see 2,3,4,7,8-pncdf is identified as the top positive effect in 3 of the folds, 2 of the folds estimate a different chemical as the top positive effect; therefore, the estimates are inconsistent. Likewise, the estimates for the top inverse association with LTL were also inconsistent across the folds. The top synergistic and antagonistic interactions were also inconsistent and, therefore, indicate no interaction in the mixed POP exposure.

## 7 Software

We have developed the InterXshift R package to implement the proposed methodology for the semiparametric discovery and estimation of interaction effects in mixed exposures using stochastic interventions. The package is accompanied by comprehensive documentation, which includes a detailed vignette elucidating the underlying semiparametric theory, practical application examples, and comparative analyses with existing methodologies. This ensures that researchers can accurately replicate and extend our analyses.

InterXshift is designed with computational efficiency in mind, optimized to operate effectively on standard personal computing environments while retaining scalability for high-performance computing platforms necessary for extensive simulations and large-scale data analyses. The package offers flexible functionalities for density estimation, interaction discovery, and TMLE, thereby allowing users to customize analyses to their specific research requirements.

Furthermore, InterXshift incorporates parallel processing capabilities to improve computational speed, which is particularly advantageous for performing simulations and cross-validation procedures inherent in our methodology. Rigorous testing has been conducted to ensure the robustness and reliability of the package, adhering to best practices in statistical software development [32].

Researchers can access the InterXshift package through our GitHub repository.^1^ The repository provides installation instructions, usage guidelines, example scripts demonstrating typical workflows, and a suite of unit tests to verify functionality and ensure reproducibility of results. By offering an accessible, well-documented, and robust statistical tool, InterXshift facilitates the application of advanced semiparametric methods in the analysis of complex exposure mixtures within the domain of causal inference and environmental health research.

## 8 Discussion and conclusion

InterXshift offers a new semiparametric perspective on detecting and estimating interactions among complex mixtures of environmental exposures. At its core is a model-agnostic causal interaction parameter that draws on stochastic shift interventions, extending earlier work on continuous treatments [17]. By defining interaction as the deviation from additivity in marginal and joint shifts, our framework resonates with traditional RERI-based approaches [1, 2] while providing greater flexibility for continuous exposures.

A central contribution of our work is the explicit derivation of the efficient influence function for interactions in vector-valued exposures. Building on this, we implement a cross-validated targeted maximum likelihood estimation (CV-TMLE) procedure that guards against overfitting and maintains asymptotic efficiency, even when numerous exposures are modeled. The CV-based discovery stage pinpoints which pairs (or subsets) of exposures exhibit synergy or antagonism, while the estimation stage then applies TMLE to quantify these interactions. Through this two-step process, InterXshift helps researchers focus on the most consequential pairwise interactions without incurring a large multiple-testing burden.

Empirically, our simulations underscore several appealing properties of InterXshift. First, we observe that the method consistently identifies true synergy or antagonism, often with high accuracy and convergence toward negligible bias under moderate sample sizes. Even in scenarios with correlated exposures and potentially limited sample size, the framework demonstrates encouraging robustness. The application to the NIEHS synthetic mixture data further illustrates how InterXshift can successfully uncover known additive and supra-additive effects, rejecting spurious signals introduced by correlated but causally inert exposures. Such strong performance suggests that this method may be applicable to a range of real-world mixture settings, where interactions are often poorly understood.

From a theoretical perspective, the shift-based definition of interaction offers a transparent causal interpretation. By conceptualizing an intervention that partially reduces or augments specific exposures, researchers can link findings to practical regulatory questions (e.g., “What if we reduced chemical A by 20% and chemical B by 10% across the population – would there be a super-additive health benefit relative to each reduction alone?”). In this way, InterXshift moves beyond merely detecting product terms in a regression and instead aligns with policy-relevant questions about actual exposure modifications.

Despite these strengths, certain limitations merit attention. The computational overhead of repeated density estimation – particularly in high-dimensional mixtures – can be substantial, and further methodological improvements are needed to ensure reliability when exposures are extremely numerous. Similarly, the method largely centers on pairwise interactions; although we show how it might extend to three- or higher-order interactions, such expansions require combinatorial increases in the number of stochastic shifts and more elaborate positivity diagnostics. Another challenge lies in mitigating positivity violations: if the shift pushes exposures outside their observed support, ratio-based estimators may exhibit inflated variance or bias. While we implement data-adaptive shrinking of the shift parameter and classification-based reparameterization to mitigate these issues, they remain points for careful monitoring in practice.

In ongoing work, we envision multiple avenues to refine and generalize the InterXshift framework. One priority is a thorough comparison of “direct targeting” and “separate estimation” approaches for the interaction parameter, including further simulations with varied density-estimation techniques. Another is to incorporate advanced neural-network or energy-balancing methods [28, 29] for ratio estimation, potentially enhancing performance under sparse or high-dimensional exposures. Additional efforts might target broader applications to longitudinal data, exploring how partial exposure shifts unfold over time, or to larger population-based datasets where interactions could be modest but still epidemiologically meaningful.

## 9 Conclusions

By merging a flexible additive-interaction definition with stochastic interventions and modern machine learning, InterXshift provides a principled way to investigate synergy and antagonism in multi-exposure data. Empirical findings in both simulations and synthetic mixtures illustrate its potential to uncover important joint effects that might otherwise remain hidden under traditional modeling. As environmental health increasingly confronts mixtures of correlated chemicals, precisely identifying synergistic hazards can guide policy to prioritize co-reductions in exposures with the greatest protective effect. We believe that, with continued methodological refinements and expanded computational strategies, InterXshift will help move the field of causal inference toward more nuanced, data-adaptive analyses of complex exposures and lead to more informed decision-making in both research and regulatory contexts.

## Figures and Tables

**Table 1:** NIEHS synthetic data interactions.

Variables	Interaction type
A1 and A2	Toxic equivalency factor, a special case of concentration addition (both increase Y)
A1 and A4	Competitive antagonism (similarly for A2 and A4)
A1 and A5	Competitive antagonism (similarly for A2 and A4)
A1 and A7	Supra-additive (“synergy”) (similarly for A2 and A7)
A4 and A5	Toxic equivalency factor, a type of concentration addition (both decrease y)
A4 and A7	Antagonism (unusual kind) (similarly for A5 and A7)

**Table 2:** Rank 1 positive marginal results for NIEHS data.

	Condition	𝚿	Variance	SE	Lower CI	Upper CI	P-value	Fold	N	Delta
1	A1	20.66	0.45	0.67	19.35	21.98	0.00	1	167	1.00
2	A1	−2.66	1.28	1.13	−4.88	−0.44	0.02	2	167	1.00
3	A1	19.13	0.53	0.73	17.70	20.56	0.00	3	166	1.00
4	Rank 1 Pos	16.31	0.14	0.37	15.59	17.04	0.00	Pooled TMLE	500	1.00

**Table 3:** Rank 1 negative marginal results for NIEHS data.

	Condition	𝚿	Variance	SE	Lower CI	Upper CI	P-value	Fold	N	Delta
1	A5	−3.94	0.70	0.83	−5.57	−2.30	0.00	1	167	1.00
2	A5	−3.27	0.87	0.93	−5.10	−1.45	0.00	2	167	1.00
3	A5	−3.50	0.61	0.78	−5.04	−1.97	0.00	3	166	1.00
4	Rank 1 Neg	−3.67	0.25	0.50	−4.65	−2.70	0.00	Pooled TMLE	500	1.00

**Table 4:** Rank 1 synergistic interaction results for NIEHS data.

	Rank	𝚿	Variance	SE	Lower CI	Upper CI	P-value	Fold	N	Delta 1	Delta 2
1	1	19.94	0.44	0.66	18.64	21.25	0.00	1	167	1.00	A1
2	1	2.91	0.84	0.92	1.11	4.71	0.00	1	167	1.00	A7
3	1	25.74	2.46	1.57	21.9	26.2	0.00	1	167	1.00	A1–A7
4	1	2.89	2.03	1.43	1.39	5.30	0.00	1	167	1.00	Interaction
5	1	−2.81	1.33	1.16	−5.07	−0.54	0.01	2	167	1.00	A1
6	1	3.23	0.80	0.89	1.48	4.98	0.00	2	167	1.00	A7
7	1	24.35	0.62	0.78	22.81	25.89	0.00	2	167	1.00	A1–A7
8	1	23.93	5.03	2.24	19.53	28.32	0.00	2	167	1.00	Interaction
9	1	3.11	0.77	0.88	1.39	4.83	0.00	3	166	1.00	A2
10	1	3.72	0.44	0.66	2.42	5.02	0.00	3	166	1.00	A7
11	1	14.46	4.50	2.12	10.30	18.62	0.00	3	166	1.00	A2–A7
12	1	7.62	3.23	1.80	4.10	11.15	0.00	3	166	1.00	Interaction

**Table 5:** Pooled TMLE estimates for rank 1 synergy in NIEHS data.

	Rank	𝚿	Variance	SE	Lower CI	Upper CI	P-value	Fold	N	Delta 1	Delta 2
1	Rank 1	6.68	0.20	0.45	5.81	7.56	0.00	Pooled TMLE	500	1.00	Var 1
2	Rank 1	3.27	0.26	0.51	2.27	4.27	0.00	Pooled TMLE	500	1.00	Var 2
3	Rank 1	21.04	0.32	0.57	19.92	22.15	0.00	Pooled TMLE	500	1.00	Joint
4	Rank 1	11.08	1.01	1.00	9.11	13.05	0.00	Pooled TMLE	500	1.00	Interaction

**Table 6:** K-fold specific antagonistic rank 1 results for NIEHS data.

	Rank	𝚿	Variance	SE	Lower CI	Upper CI	P-value	Fold	N	Delta	Type
1	1	−3.97	0.71	0.84	−5.62	−2.32	0.00	1	167	1.00	X5
2	1	3.00	0.84	0.91	1.21	4.79	0.00	1	167	1.00	X7
3	1	−1.76	1.00	1.00	−3.72	0.20	0.08	1	167	1.00	X5–X7
4	1	−0.78	0.93	0.96	−2.67	1.11	0.42	1	167	1.00	Interaction
5	1	−3.48	0.74	0.86	−5.16	−1.79	0.00	2	167	1.00	X5
6	1	3.21	0.80	0.89	1.46	4.96	0.00	2	167	1.00	X7
7	1	−0.61	1.22	1.10	−2.77	1.56	0.56	2	167	1.00	X5–X7
8	1	−0.34	1.08	1.04	−2.38	1.70	0.74	2	167	1.00	Interaction
9	1	−3.45	0.54	0.73	−4.89	−2.01	0.00	3	166	1.00	X5
10	1	3.83	0.43	0.66	2.54	5.13	0.00	3	166	1.00	X7
11	1	−0.69	1.17	1.08	−2.81	1.43	0.50	3	166	1.00	X5–X7
12	1	−1.07	0.90	0.95	−2.94	0.79	0.27	3	166	1.00	Interaction

**Table 7:** Quantile G-Computation interaction results from NIEHS synthetic data.

	Estimate	Std. Error	Lower CI	Upper CI	Pr(>|t|)
(Intercept)	21.29	1.58	18.19	24.39	0.00
psi1	0.02	1.62	−3.16	3.20	0.99
psi2	0.59	0.67	−0.71	1.90	0.37

**Table 8:** Effects selected by glinternet.

Main effects	
Variable	Coefficient
A1	11.722
A2	4.369
A3	3.004
A4	−1.547
A5	−3.225
A6	−0.211
A7	3.327
Interaction effects	
Variables	Coefficient
A1 × A2	−2.092
A1 × A3	−1.328
A2 × A7	0.625
A5 × A7	−0.509
A4 × A5	0.425
A2 × A3	0.350
A2 × A6	−0.259
A1 × A7	−0.182
A3 × A5	−0.179
A2 × A4	0.150
A5 × A6	0.142
A6 × A7	−0.122
A4 × A7	−0.105

**Table 9:** Rank 1 results for different conditions.

	Condition	Psi	Variance	SE	Lower CI	Upper CI	P-value	Fold	N	Delta
1	2,3,4,7,8-pncdf	0.02	0.00	0.02	−0.02	0.05	0.42	1	202	2.88
2	2,3,4,7,8-pncdf	0.01	0.00	0.02	−0.03	0.04	0.64	2	202	2.88
3	PCB74	−0.05	0.00	0.03	−0.11	0.02	0.18	3	201	6,794.89
4	1,2,3,4,7,8-hxcdf	0.00	0.00	0.02	−0.04	0.05	0.92	4	201	2.49
5	2,3,4,7,8-pncdf	0.03	0.00	0.02	−0.01	0.08	0.12	5	201	2.88
6	Rank 1	−0.00	0.00	0.01	−0.03	0.02	0.74	Pooled TMLE	1,007	1,361.20

## Footnotes

1 https://github.com/blind-contours/InterXshift.

## A Density estimation of the exposures

Estimating the density of each exposure is crucial for evaluating the effects of interventions both on average across the population and within subpopulations. Accurate density estimation ensures that the stochastic interventions are properly modeled and that the subsequent causal inferences are valid. In this appendix, we provide a detailed exposition of the two approaches to density estimation employed in our methodology: Direct Density Estimation and Reparameterization of Density Estimation via Classification. Additionally, we delve deeper into the theoretical underpinnings, implementation nuances, and practical considerations for each method.

### A.1 Direct density estimation

Direct density estimation involves modeling the conditional density of the exposures given the covariates directly. This approach can be challenging, especially in high-dimensional settings, due to the complexity of accurately capturing the joint distribution of multiple exposures. To address this, we employ advanced machine learning techniques that can flexibly model complex relationships.

#### A.1.1 Super learner algorithm

The Super Learner (SL) algorithm is an ensemble method that combines predictions from a diverse library of base learners to optimize predictive performance. The SL operates in two primary steps:

1. **Base Learners Training:** Multiple base learners (e.g., generalized linear models, random forests, gradient boosting machines) are trained on the data. Each base learner captures different aspects of the data’s underlying structure.
2. **Meta-Learner Aggregation:** The predictions from the base learners are combined using a meta-learner. In our implementation, we utilize a Non-Negative Least Squares (NNLS) approach to assign optimal weights to each base learner based on their performance, ensuring that the combined prediction leverages the strengths of each individual model.

This ensemble approach enhances the robustness and accuracy of density estimates by mitigating the biases inherent in individual models.

#### A.1.2 Homoscedastic errors (HOSE) learner

The HOSE learner assumes that the variance of the errors is constant across all levels of the predictors, an assumption known as homoscedasticity. Under this assumption, the HOSE learner employs a semi-parametric approach to density estimation:

1. **Mean Estimation:** Similar to the Super Learner, a diverse set of base learners is used to estimate the mean function μ(A,W)=E[Y∣A,W].
2. **Error Variance Modeling:** Given the homoscedasticity assumption, the variance of the errors is estimated uniformly across all observations.

This approach simplifies the density estimation process by reducing the complexity associated with varying error structures, making it computationally efficient for certain datasets.

#### A.1.3 Heteroscedastic errors (HESE) learner

Contrary to the HOSE learner, the HESE learner allows for varying error variances across different levels of the predictors, accommodating heteroscedasticity. This method employs a two-step semi-parametric approach:

1. **Mean Estimation:** As with the HOSE learner, a Super Learner is used to estimate the mean function μ(A,W)=E[Y∣A,W].
2. **Variance Estimation:** An additional Super Learner is employed to model the variance of the errors, $\sigma^{2}(\text{A},\text{W})=\text{Var}(Y|\text{A},\text{W})$, allowing the error variance to adapt based on the values of the predictors.

The HESE learner provides a more flexible and accurate density estimation in scenarios where the assumption of constant variance is violated, albeit at the cost of increased computational complexity.

### A.2 Reparameterization of density estimation via classification

As an alternative to direct density estimation, we propose a novel approach that redefines the problem of estimating the density ratio H(A,W) as a classification problem in an augmented dataset. This method leverages the fact that classification algorithms are well-developed and widely available in the machine learning literature compared to density estimators. This approach is used in other methodologies, such as in longitudinal modified treatment policies [33].

To apply this approach, we construct an augmented dataset consisting of duplicated observations. For each original observation, we create a duplicate with the observed exposures A and another with the exposures under the intervention ${\text{A}}^{\delta }$. We introduce an indicator variable ξ, which is set to 1 if the duplicated observation corresponds to the exposure under intervention, and 0 otherwise. Here, exposure can be the intervention on multiple exposures, such as the joint shift we need in our interaction parameter. The augmented dataset can be represented as:

$$\left\{\left(W_{\xi ,i,t},{\text{A}}_{\xi ,i,t},\xi_{\xi ,i}\right):\xi =0,1;i=1,\ldots ,n\right\},$$

where $\xi_{\xi ,i}=\xi$ indexes the duplicates, ${\text{W}}_{\xi ,i,t}={\text{W}}_{i,t}$ is the history variable, and ${\text{A}}_{\xi ,i,t}=\xi \cdot {\text{A}}_{i,t}^{\delta }+(1-\xi )\cdot {\text{A}}_{i,t}$ is the natural exposure level if ξ=0, and the intervened exposure level if ξ=1.

We denote the probability distribution of $\left({\text{W}}_{t},{\text{A}}_{t},\xi \right)$ in the augmented dataset by $P_{\xi }$, with corresponding density $p_{\xi }$. We define the parameter $u_{\xi }$ of $P_{\xi }$ as:

$$u_{\xi }\left({\text{a}}_{t},{\text{w}}_{t}\right)=P_{\xi }\left(\xi =1|{\text{A}}_{t}={\text{a}}_{t},{\text{W}}_{t}={\text{w}}_{\text{t}}\right).$$

The density ratio $H_{t}\left({\text{a}}_{t},w_{t}\right)$ can be expressed in terms of $u_{\xi }$ as follows:

$$H_{t}\left({\text{a}}_{t},{\text{w}}_{t}\right)=\frac{p_{\xi }\left({\text{a}}_{t},{\text{w}}_{t}|\xi =1\right)}{p_{\xi }\left({\text{a}}_{t},{\text{w}}_{t}|\xi =0\right)}=\frac{P_{\xi }\left(\xi =1|{\text{A}}_{t}={\text{a}}_{t},{\text{W}}_{t}={\text{w}}_{t}\right)}{P_{\xi }\left(\xi =0|{\text{A}}_{t}={\text{a}}_{t},{\text{W}}_{\text{t}}={\text{w}}_{\text{t}}\right)}\;=\frac{u_{\xi }\left({\text{a}}_{t},{\text{w}}_{t}\right)}{1-u_{\xi }\left({\text{a}}_{t},{\text{w}}_{\text{t}}\right)},$$

where the first equality follows from the definition of $H_{t}$ and the definition of conditional density, the second from Bayes’ rule and the observation that $P_{\xi }(\xi =1)=P_{\xi }(\xi =0)=\frac{1}{2}$, and the third from the definition of $u_{\xi }$.

By estimating $u_{\xi }$ in the augmented dataset using any classification method available in the machine learning and statistical learning literature (e.g., Super Learner), we can directly estimate the density ratio. This classification-based approach ensures that the estimator retains desirable properties such as asymptotic normality, provided that appropriate cross-validation and cross-fitting techniques are employed. Furthermore, this reclassification approach is substantially faster computationally, which is necessary, especially in our proposed methodology, where the conditional density is needed for each exposure and higher folds in cross-validation provide more consistent results. Overall, this approach has been used in previous literature to avoid density estimation. We simply create a duplicate dataset, shift exposures by δ, and create an intervention outcome = 1 for this dataset. Bind rows with our unperturbed data with the outcome intervention = 0. Then we train a classifier and use Bayes’ rule to estimate the density ratio comparing shift to no shift.

In our implementation, users can choose between direct density estimation and the reparameterization approach via a user-defined parameter. Moving forward, all applications and simulations use the classification approach.
